# The Value of Sulphur in the Treatment and Prevention of Malaria

**Published:** 1907-07

**Authors:** 


### NOISES.

Every season brings its joys and woes to the urban dweller. It is likely that the rural sojourner also has his little troubles, and God does not always temper the wind to the shorn lamb, so that those who live in cities, especially on the outskirts thereof, find themselves participating in the grievances and annoyances of both country and city life.

Winter has its drawbacks, but possesses one cardinal advantage over summer, in that during the former season windows are closed. The cock's shrill clarion does not disturb our matudinal slumbers nor rouse us from our lowly bed, and the rattle of the coon-driven dray without has the pianissimo stop put on by the protecting window and becomes merely a vanishing arythmic purr in the distance. But for the human voice the stillness of the office is broken only by the sibillation of the radiator and the thump of the patient's jaw as it strikes the floor when the amount of his bill is sprung on him.

These soft immunities from noises are thrown to the wind when the first burst of summer sends the windows up with a rush. Then what a change takes place. No longer is the morning rest serene and unbroken, but through the open window at the peep of dawn comes the defiant challenge from your neighbor's rooster to meet instant response from your own proud Sir Chanticleer, (if you have not outlived the city dweller's fondness for precarious poultry raising)and that to be taken up seriatim far and near as each feathered champion stalks into the field. What remnant of repose remains is torn away by the barking of the dog at the milkman› the imperious ring of the early vegetable huckster, the ambitious young musician practicing her scales and a near-by neighbor trying a few new records on the phonograph. Then you arise with a groan, scrape the bristles from your face, give a perfunctory dab at your hands and neck and finally hie you to the marts of commerce.

If you have the misfortune to be a practitioner of the healing art, your troubles have but just begun. In your office, there is no longer found the chloistral stillness of the winter, but the palpitating air vibrates with all manner of discordant noises. Ausculation and percussion are vain and futile and conversation must be maintained in a tone of voice better suited to the bosum of a whaling barque than a grave physician engaged in inspiring confidence in a new patient.

The insistent din of the electric theatre, the roar of the passing dray hurried over rough stones, the whirr of the auto, shouts and cries, form a medley of sounds which paralyzes the mental functions and makes thought flow as smoothly as a flat-wheeled car.

There may be those phlegmatic, boiler-maker sort of people who are never happy unless they are in a vortex of sound but there are few circumstances connected with city life which are more wearing on a nervous man than unnecessary, unmitigated and unavenged noises. A great desire surges up in the soul of the tortured to be possessed of a club on the order of a lazy rack which he might project out of the window and sweep the nigger draymen off of his seat as you would flick a May fly from a horse's rump. There is nothing so irritating as the smug serenity of this individual sitting like a sort of black Kraken in his Maelstrom of noise. Cities are built and civic affairs are carried on with a view to the requirements of the normal man and the neurasthenic though respectable in number has no consideration paid him. A bill should be introduced into the present general assembly that displays such a frenzied zeal for reform which would prohibit roosters from crowing before eight in the morning, banish pianos and phonographs, cut the tongues out of newsboys, the hoofs off of horses deport all negroes to the Gaboon, put rubber tires on every vehicle and pave the streets with art squares. The passage of this safe and sane bill would give some ease of life to the neurasthenic and yield him a measure of that peace which he seems doomed never to know on earth.

